# Semantic modelling of animal welfare explained – Part 1: Calculating overall welfare scores for husbandry systems using the ANyWEL model framework

**DOI:** 10.5194/aab-69-363-2026

**Published:** 2026-07-16

**Authors:** Janine Benthin, Karen Kauselmann, E. Tobias Krause, Marc B. M. Bracke, Margret L. Vonholdt-Wenker

**Affiliations:** 1 Institute of Animal Welfare and Animal Husbandry, Friedrich-Loeffler-Institute, 29223, Celle, Germany; 2 Wageningen Livestock Research, Wageningen University & Research, Wageningen, 6700 AH, the Netherlands

## Abstract

A modern evidence-driven approach to assess the welfare of farm animals is semantic modelling. It enables the (semi-)quantitative assessment of the overall welfare status, as well as a welfare comparison of different housing systems, through systematic and formalised knowledge extraction from the scientific literature. A semantic model essentially enables a description of housing systems in terms of their welfare-relevant properties (so-called attributes, e.g. space per pen or the availability of enrichment materials) using weighted welfare component scales. Based on this approach, we developed the ANyWEL framework (ANimal WELfare assessment of anY farm animal): a semantic-modelling framework to assess the welfare status of any species of farmed animal kept in housing systems that meet predefined criteria (i.e. the model's assessment domain and the available scientific literature). Compared to previous semantic models, the ANyWEL framework is not restricted to a single species or production direction. It is generalised across species and production directions. Due to its conceptual character, the ANyWEL framework can be applied to new species and systems in the future. We show that it can use new scientific knowledge, even when this would go against mainstream perceptions (e.g. that extensive systems “must be” better for welfare than intensive systems). The objectives of this paper are (i) to explain the general procedure and principles used in semantic modelling, (ii) to introduce the ANyWEL framework by comparing it with a previous semantic model and to illustrate it with an example of a fictitious animal species called anYmal, and (iii) to reflect on the strengths and limitations of this new generalised model framework even with unexpected outcomes.

## 1 Introduction

Public concern about farm animal welfare is increasing (European Commission, 2023). A growing public demand for higher welfare standards puts pressure on policy-makers, farmers, and scientists to establish more welfare-friendly housing systems (Alonso et al., 2020; European Commission, 2022; Keeling et al., 2022). Concern for farm animal welfare is part of the increasingly urgent need to develop food systems and societies that are sustainable overall, environmentally and socially, as well as economically. To support the societal demand for a more sustainable food system, a valid, reliable, and transparent evaluation procedure is called for to facilitate the assessment of livestock welfare in different housing systems based on the available scientific information. Direct “measurement(s)” of animal-based features have been used by scientists (e.g. qualitative behavioural assessment (QBA, e.g. Cooper and Wemelsfelder, 2020), judgement/attention bias tests (Jones et al., 2018), and Welfare Quality^®^ (Blokhuis et al., 2010)), with their respective strengths and weaknesses. Nevertheless, a science-based overall farm animal welfare assessment remains a major challenge that, in the past, has been addressed mainly using expert surveys and panels focussing on expert consensus (Anonymous, 2001; EFSA, 2007, 2010, 2012, 2015, 2023; Bracke et al., 2008a, b). A more formalised scientific method that makes more explicit use of the numerous studies that have been conducted over the past decades would be beneficial to assess overall welfare (Heinonen et al., 2013; Blanco-Penedo et al., 2020; Verdon, 2022). Besides this, the rapid developments in artificial intelligence (large language models) may increasingly allow for automated modelling (Chowdhary, 2020; Reimers and Gurevych, 2019; Qin et al., 2024; Zhang et al., 2026). Enabling overall assessments of animal welfare is also called for by the growing need for integrated sustainability assessments, where animal welfare to date is only a component besides other social, environmental, and economical components (Bracke et al., 2023). In order to provide a decision support tool that could synthesise the available scientific knowledge about farm animal welfare, a formalised and systematic procedure called semantic modelling of animal welfare has been developed. This procedure can, in principle, incorporate all relevant scientific studies obtained in a broad literature review (Bracke, 2001, 2008; Bracke et al., 2008a).

Semantic modelling is a method to systematically extract and analyse statements (quotes) from the scientific literature that describe how animal welfare is affected under different housing conditions in order to generate an overall welfare score. The term “overall” is used here to denote that all aspects (i.e. all welfare needs of the animals) are considered, as well as all available scientific information (i.e. all relevant and meaningful scientific statements). Compared to a meta-analysis or systematic review, semantic modelling does not require as detailed quantitative reports of, for example, effect sizes or similar numerical and statistical parameters, which often are not systematically reported. Instead, semantic modelling procedures enable a data extraction from the scientific literature using a decomposition of the scientific statements based on an understanding of their meaning in the field of welfare science. Through modelling, written scientific statements are systematically converted into welfare-relevant attributes of housing systems. Different housing systems consist of attributes that can each have an effect on the welfare of animals. Examples of welfare-relevant attributes may include the type of floor, water provision system, and lighting system. Each of those attributes have so-called attribute levels. So, for instance, the water provision system can exist out of drinking nipples, open bowls, or troughs. The attributes receive their weighting factors and so-called attribute level scores based on the science-based ranking of the levels of each attribute in the welfare model (see Table 1 for a glossary). The model takes a description of a housing system as input to produce a welfare score calculated as a weighted average of the attribute level scores and the weighting factors of the attributes (welfare indicator variables) that make up the model. The definition and specification of the welfare-relevant attributes in a semantic model depend on the meaning and systematic decomposition of the scientific statements collected in the database. So, semantic modelling is a method to assess descriptively the overall welfare state as a score on a scale (e.g. 0–1 or, in previous models, 0–10). This is done in a formalised way for a type of (farm, zoo, wild, or pet) animal over a period of time in an assessment domain. The method is based on scientific knowledge with the objective to improve moral and political decision making and sustainable development. In semantic modelling, welfare is typically defined as the quality of life as perceived by the animal itself (Bracke et al., 1999), i.e. as the degree of satisfaction and frustration (i.e. positive and negative affect) of the animal's welfare needs. These needs reflect the state of the animal's cognitive–emotional control systems (which roughly correspond to its behavioural systems or life functions). The overall welfare score (OWSc; see Table 1) estimates, based on available science, the actual welfare state of the animals in a given housing (and management) system in relation to what is theoretically possible. Thus far, semantic models for farm animal welfare assessment have been made for various species and/or specific production directions, such as pregnant sows (SOWEL, Bracke et al., 2002a, b), laying hens (FOWEL, De Mol et al., 2006; Shimmura et al., 2011), dairy cows (COWEL, Ursinus et al., 2009), Atlantic salmon (SWIM, Stien et al., 2013; Pettersen et al., 2014; Folkedal et al., 2016), and rainbow trout and pikeperch (Tschirren et al., 2021). In addition, semantic models have been constructed to assess enrichment materials for pigs (RICHPIG, Bracke, 2008), to assess the risk of tail biting in pigs (PIGTAIL, Bracke et al., 2004a, b), and to assess the severity of handling (esp. sea lice treatments) in Atlantic salmon (Helge Stien et al., 2025). Finally, semantic modelling principles have been used to assess the welfare of calves (Bracke et al., 2008b) and broilers (Bracke et al., 2019) at the system level, the need for wallowing behaviour in pigs (Bracke and Spoolder, 2011), and cross-species comparisons of animal welfare (Bracke, 2006).

**Table 1:** Glossary – explanation of important terms used in semantic modelling to assess animal welfare.

Term	Explanation
Assessment domain	The set of elements (i.e. systems) covering the whole range of housing (and management) systems the model is designed to assess such that a welfare score can be calculated for all systems in the assessment domain. The assessment domain is comparable to the boundaries set in a life cycle analysis. The assessment domain is delineated by the type of animal (e.g. species and age class or reproductive state), the range of living environments of the animal (e.g. entire housing and management systems or a subset of housing systems, e.g. only systems with good management practices), and the aspects of welfare covered by the model (e.g. related to the definition of welfare and its decomposition in terms of welfare needs; see below).
Attribute	Variable used to specify a welfare-relevant characteristic or property of a housing system (e.g. space allowance, group size, enrichment material), also called a welfare indicator. An attribute in a welfare model has two or more attribute levels (see below) that, together, cover the entire assessment domain. Together, the attributes in the model cover all welfare needs (e.g. the attribute of group size addresses the need for social contact; the attribute of space allowance addresses the need to be able to move around).
Attribute level	Welfare-relevant property of a case or element (e.g. housing system) in the assessment domain. For example, the attribute of group size may have as its levels “1”, “2–3”, and “4–6 animals”; the attribute of space allowance may have as its levels “0.5–0.8 $m^{2}\;{\mathrm{animal}}^{-1}$ ” and “ >0.8 –1.5 $m^{2}\;{\mathrm{animal}}^{-1}$ ”. The identification of different levels of an attribute must have a scientific basis; i.e. attribute levels are ranked for welfare (from worst to best) based on scientific statements from studies reporting (behavioural; stress – physiological or pathological) welfare effects between the different levels. This is related to the fact that two (or more) experimental treatments show a significant effect on some welfare measure. Together the attribute levels of each attribute in the model must cover the entire assessment domain (i.e. cover all welfare-relevant properties) of all possible housing systems that the model is designed to be able to assess. An overall welfare score (OWSc) for a housing system can be calculated only if, for each attribute in the model, exactly one attribute level has been selected to describe that housing system.
Attribute level score (ALevSc)	A score (e.g. between 0 and 1) for each of the levels of an attribute in the model. These levels are based on the underlying scientific statements reporting welfare effects, thus allowing the attribute levels to be ranked for welfare. Based on this ranking, the best attribute level can, for example, receive a score of 1, and the worst can receive a score of 0, and all intermediate attribute levels can get intermediate ALevScs proportionally to their ranks (alternatively, they can get a differentiated score based on the weightings using the weighting categories as implemented in the FOWEL and COWEL models (De Mol et al., 2006; Ursinus et al., 2009); see also below).
Housing (and management) system	These are the environmental conditions in which (farm) animals are living, to be described in terms of the welfare attributes and their levels. In previous models, e.g. SOWEL (Bracke et al., 2002b), the term “housing and management system” was used to denote the animals' living environment, which included not only the system hardware (buildings, feeders, drinkers, pen fittings, etc.) but also the animals' social environment (e.g. group size) and aspects of the environment typically provided by the farmer (i.e. management conditions), hence the term housing and management system. The assessment domain of the ANyWEL model framework, however, was developed focusing on system hardware (structural technical elements) of commercial systems for livestock assuming good management conditions (i.e. management that is in accordance with what are generally considered to be good practices). The ANyWEL model framework itself, however, is not per se restricted to that; i.e. it allows for the addition of the whole range of suboptimal management aspects of the system into the modelling procedure, provided there is a sufficient scientific basis to do so. The ANyWEL framework can also be applied beyond farm animals, e.g. to pets and laboratory animals.
Overall welfare score (OWSc)	Welfare score of a housing system that expresses the degree to which all of the animal's welfare needs are satisfied or frustrated. The OWSc can range, for example, between 0 and 1 (worst to best). The OWSc is calculated in the ANyWEL model framework, as well as in SOWEL (Bracke et al., 2002b), as the weighted average of all attribute level scores of a housing and management system.

**Table 1:** Continued.

Term	Explanation
Scientific statement	This is the section of the scientific literature that describes a scientifically meaningful finding. This is typically a statistically significant difference between two (or more) treatments (or factors, also called dependent variables, which are typically environment-based variables) as measured by some welfare measure (also called the independent variable, which is typically a behaviour, health or (stress-related) physiological parameter and, hence, is an animal-based variable). The following is an example: “A choice test showed that anYmals (i.e. our fictitious animal species used to illustrate the ANyWEL model framework see below) highly preferred ropes over balls and balls over chains”. The welfare input is an environment which provides enrichment material (ropes, balls, chains) so that the animals can express their need for exploration. The welfare output is the observation that anYmals show clear preferences. The attribute here is enrichment material. It has three attribute levels, namely rope, ball, and chain. Here, the attribute level of rope means that a rope is being provided in a housing system. If this is the case, then such systems are better for anYmal welfare than systems where a ball or a chain is provided, other things being equal (and given that this exemplary scientific statement is true and indeed expresses what anYmals prefer).
Semantic modelling	This is a formalised procedure that aims to provide a quantified assessment of animal welfare based on the scientific literature. A decisive role in the translation (systematic use) of scientific statements collected in the database for the assessment of overall animal welfare in a housing system in the assessment domain is played, in particular, by the understanding of the scientific meaning of words and phrases used in the scientific statements, hence the term “semantic modelling of animal welfare”. In the example statement above (of anYmals highly preferring ropes over balls), the words “prefer” and “highly” are to be interpreted in a scientific context. This means, for example, that anYmals presumably had a voluntary choice to interact with these materials and that the experimental design controlled for other factors that could explain the observed difference. Furthermore, there presumably was a scientifically sound reason to use the word “highly”, e.g. implying a large effect or elevated motivational strength as indicated by a willingness to work for the resource.
Type of welfare measure	This refers to a specific (usually animal-based) welfare measure, e.g. time spent interacting with an enrichment material, lameness prevalence, or number of skin lesions. The term “type” is used to distinguish different types of welfare measures within weighting categories (WCat; see below). That is, a welfare measure is first classified as belonging to a certain WCat, and within that WCat different types of measures are labelled as different types. Their function in the model is to add a bit of welfare weight that is generated in the first place by the WCat. For example, the WCat of pain covers different measures indicative of pain, labelled in the model as different types (e.g. lameness prevalence and number of skin lesions are two types of measures in the WCat of pain). We avoid the use of the term “welfare indicator” to denote welfare measures because the term has been used in the SWIM models (Stien et al., 2013; Pettersen et al., 2014) to refer to attributes.
Weighting category (WCat)	This is the class of welfare measures belonging to a welfare measurement paradigm, related to the scientific fields of (applied) ethology, (evolutionary) biology, pathology, and (stress-related) physiology. Some of the WCats or classes of (types of) welfare measures indicate negative welfare (e.g. illness, abnormal behaviour, aggression, frustration and avoidance), while other WCats imply positive welfare (namely, the WCats of demand, preferences, and natural behaviour). WCats are used to calculate weighting factors (WFs) of welfare-relevant attributes in the model. Some WCats have a relatively low impact on welfare, while others have a relatively high impact (see below, and see Vonholdt-Wenker et al., 2026).
Weighting-category level score (WCatLevSc)	Each welfare measure that differentiates between two (or more) attribute levels has a certain magnitude, referring to the intensity, duration, and incidence of the welfare impact. WCats can be distinguished as positive and negative WCats and as WCats that have a relatively low or high impact on welfare. The former distinction generates a WCatLevSc with either a positive or a negative sign, while the latter distinction originally (in previous semantic models) generates WCatLevScs on either a three- or a five-point scale, each with three levels ( ±1 , 2, and 3 or ±1 , 3, and 5). In the ANyWEL framework we added several points on these scales, namely 0, 0.01, 0.25, 0.4, and 0.5 (see below). For each welfare-relevant attribute in the model the maximum WCatLevScs of each positive WCat and the minimum WCatLevScs of each negative WCat are added for the best and worst attribute level, respectively, thereby generating the basis to calculate a WF for each attribute (see below).

**Table 1:** Continued.

Term	Explanation
Weighting factor (WF)	This is the calculated score for each attribute that (roughly) indicates how much that attribute contributes to overall welfare compared to the other attributes in the model. The WF is calculated from the diversity and biological significance of welfare effects described in the scientific statements, formally represented by the (maximum and minimum) WCatLevScs assigned to the best and worst level of each attribute and the number of different types of welfare measures within each WCat, as identified in the scientific statements. There are two types of WF: absolute WFs and relative WFs. The relative WF of an attribute is the absolute WF of that attribute divided by the sum of the absolute WFs of all attributes in the model. When the relative WFs for each attribute are multiplied by its attribute levels score (ALevSc) and then summated over all attributes, this is the overall welfare score (OWSc) for the housing system (specified by its assigned attribute levels) on a scale from 0 to 1. Alternatively, the OWSc can be calculated by multiplying the absolute WFs by the ALevScs, summating these, and then dividing this sum by the sum of the absolute WFs.
Welfare need	A welfare need is a component of overall welfare, i.e. what the animal needs for welfare, where welfare is defined as the quality of life as perceived by the animal itself (Bracke et al., 1999). Welfare is the degree of satisfaction and frustration of all the animal's welfare needs, as indicated by the state of the animal's cognitive–emotional control systems (which roughly correspond to its behavioural systems or life functions). Each need has a (strong) behavioural component but also an emotional component (feelings or affective states with positive or negative valence), as well as a (stress-related) physiological and usually also a disease-related component. For example, regarding metabolism, animals show food searching and consumption behaviour, presumed feelings of hunger and satiety, and the physiological regulation of satiety, as well as stress responses related to the ability to obtain food, and there is pathology related to starvation and obesity. Welfare needs are defined by the (presumed) presence of emotions, feelings, or affective states and are measured by behavioural, pathological, and (stress-related) physiological measures (as classified by the WCats). Welfare needs, i.e. what animals need for welfare, are to be distinguished from biological needs, i.e. that which animals need to survive and reproduce (in nature). Example welfare needs are the needs for food and water intake, thermal comfort, rest, social contact, exploration, the ability to move around, and (a perception of) safety.

In this paper, we present a semantic-modelling framework that further improves the main methodology previously developed in semantic modelling. This work is part of an applied project assessing existing housing systems in Germany (https://www.ktbl.inkalktier.de, last access: 3 July 2026) using semantic modelling on the basis of the SOWEL model. Although the basic principles of semantic modelling were described earlier and were presumed to be clear and bio-logical, we found it was necessary to further clarify and refine the rules to allow new modellers to understand and apply the state-of-art semantic-modelling methodology. This new framework is geared towards a welfare assessment of all major production directions in both existing and novel housing systems. To this end, we developed the ANyWEL (ANimal WELfare assessment of anY farm animal) framework. It can be used to calculate the overall welfare score (OWSc) of any housing system for any production direction once the current scientific knowledge base has been modelled. To this end, artificial intelligence (AI) tools are being developed (see Zhang et al., 2026). As semantic modelling may appear to be a rather abstract activity that is harder to grasp than previously thought, the objective of this paper is to explain the semantic ANyWEL model framework in more detail with the help of a fictitious species called anYmal. In addition, we evaluate the strengths of the ANyWEL model (compared to the original SOWEL model) and its potential limitations. In the companion paper (Part 2, Vonholdt-Wenker et al., 2026), we explain in more detail how scientific statements are to be decomposed formally such that attribute levels can be ranked for welfare based on the scientific information contained in the scientific statements.

## 2 Procedure of semantic modelling

**Figure 1:**
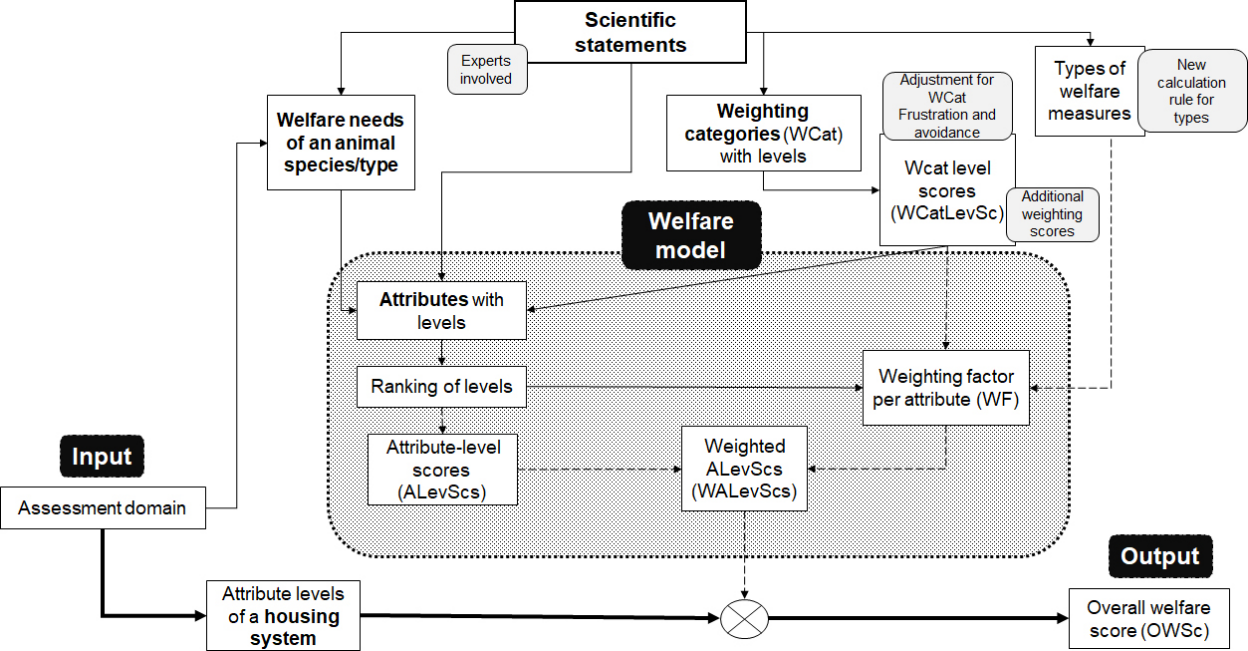
Schematic overview of the semantic ANyWEL model framework (modified after Bracke et al., 2002b). The dashed arrows indicate calculations. Solid arrows are relevant steps for semantic modelling. The grey box with a dotted line is the welfare model. Light-grey boxes with a solid line are the refinements developed for the ANyWEL model, which are explained below.

To date, semantic welfare models have mainly (but not exclusively) been developed for welfare assessment at the system level rather than, for example, at the farm level (with the SWIM models being a notable exception to this rule). Typically, semantic models consisted of a relational database that stores (i) scientific statements collected from the literature, (ii) descriptions of typical benchmark housing systems covering the assessment domain for which the model was designed using (iii) so-called welfare-relevant attributes (or welfare indicators, such as the attribute of group size), (iv) the ranked attribute levels (e.g. the values of small, medium, and large group size, which specify the welfare-relevant properties of the housing systems), and (v) the welfare needs of animals (e.g. the needs for social contact, movement, safety, thermal comfort) as a basis for the calculation of the OWSc for each housing system. The OWSc is calculated as the weighted average score of the model's attribute level scores (ALevScs, Fig. 1 and Table 1). Model attributes have weighting factors (WFs) which are derived from the scientific statements using a classification involving (vi) so-called weighting categories (WCats, which are classes of welfare measurements) and (vii) weighting-category level scores (WCatLevScs). The basis for modelling is the WCatLevSc. It essentially represents a statistically significant welfare difference between two attribute levels (one of which is better for welfare than the other). This difference generates the ALevScs and the WFs of the attributes that, in the end, make up the welfare model (Fig. 1).

To illustrate the principles of the new ANyWEL model framework, we introduce a fictitious species called anYmal. This is a simple species, kept in only three different housing conditions. The example illustrates the modelling principles with a limited number of welfare needs and model attributes. We use this simplified and imaginary example also to show how the new ANyWEL framework compares to the original SOWEL modelling rules. The illustration is intended not to empirically validate the new model but rather to illustrate how semantic modelling works and to demonstrate the added value of the refined procedures proposed in the ANyWEL framework.

### 2.1 Introducing semantic modelling using the anYmal as an artificial species with welfare needs

We establish anYmal as a newly created animal species by artificially mixing genes. So, this species does not exist in nature and is to be farmed either intensively (I), semi-intensively (S), or extensively (E). These three housing conditions vary only in terms of group size, the provision of enrichment materials, and space allowance and not, for example, with respect to food and water. All anYmals get the same amount of pelleted food (ad libitum) and good-quality drinking water (Table 2). Extensively kept anYmals have more space and are kept in smaller groups. To “enrich” the indoor environment, farmers provide chains in intensive systems and balls in semi-intensive and extensive systems (Table 3). The meat of anYmals reared in these systems is to be sold as low, medium, and high welfare, respectively. Scientists have, however, started to study anYmals in more detail and found that they are behaving differently from common farm animals. We designed this example such that it brings out the role of scientific knowledge in welfare assessment and its ability to overrule common perceptions and expectations about what (we, too, believe) is good for welfare (e.g. that extensive systems are generally better for welfare; see, for example, expert consultations reported in Bracke et al., 2002a, 2008a, and 2019, on sows, calves, and broilers, respectively). The welfare calculations will show that, in this fictitious case and in contrast to what might be expected, the extensive system is the worst for anYmal welfare because of what is (postulated to be) “known” about this fictitious species (see Table 3).

**Table 2:** Three housing systems (intensive, semi-intensive, and extensive) for the imaginary and newly created anYmal species.

Descriptive attributes	Intensive housing	Semi-intensive housing	Extensive housing
Food provision^a^	Pellets	Pellets	Pellets
Water supply^a^	Ad lib	Ad lib	Ad lib
Space allowance	1 $m^{2}\;{\mathrm{anYmal}}^{-1}$	1 $m^{2}\;{\mathrm{anYmal}}^{-1}$	5 $m^{2}\;{\mathrm{anYmal}}^{-1}$
Group size	15 anYmals	8 anYmals	3 anYmals
Enrichment material^b^	Chain	Ball	Ball

^a^ These attributes show no differences between the housing systems and so are not included in the model.^b^ Ropes have been described as enrichment material, but these are not offered in any of the three housings systems due to high costs.

**Table 3:** Fictitious scientific statements about the welfare of the imaginary species called anYmal. StatID: statement identification number.

StatID	Scientific statements per welfare-relevant attribute
A	Space allowance
1	A choice test revealed that anYmals enter more-crowded pens (stocked with 1 $m^{2}\;{\mathrm{anYmal}}^{-1}$ ) much more often than less crowed pens (stocked with $>1\;m^{2}\;{\mathrm{anYmal}}^{-1}$ ).
2	A higher frequency of abnormal mounting behaviour was observed at a space allowance of 5 $m^{2}\;{\mathrm{anYmal}}^{-1}$ compared to 1 $m^{2}\;{\mathrm{anYmal}}^{-1}$ .
B	Group size
3	An operant conditioning test showed that anYmals are highly motivated to leave groups of 3 to get access to groups of 15 anYmals. They pushed a gate of 200 kg to get away.
4	AnYmals had much more bleeding wounds from aggressive biting in groups of three compared to in groups of eight due to agonistic interactions.
5	Groups of 15 anYmals have less skin scratches compared to smaller groups.
6	AnYmals kept in a group of 3 showed a high number of bruises compared to groups of 15 anYmals, where no bruises were found.
7	Signs of severe lameness were observed in groups of three anYmals compared to in groups of eight anYmals. The latter group only showed moderate lameness incidences.
8	Groups of 3 anYmals often fight heavily, resulting in high mortality rates, compared to groups of 8 or 15 anYmals.
9	A group of 3 anYmals tended to have a higher risk for cold stress as groups of 3 anYmals seem to huddle less compared to larger groups with more than 10 anYmals.
10	AnYmals kept in groups of 3 anYmals showed a much higher number of life-threatening diseases, such as bacterial and viral respiratory infections due to a low immune response, than in groups of more than 10 anYmals.
11	Groups of 8 anYmals have less body contact to remove parasites compared to groups of 15. Hence, more problematic skin diseases are found in smaller groups.
C	Enrichment material
12	The duration of time spent playing with balls was higher than the time spent playing with chains.
13	The use of chains caused both mouth and teeth injuries, whereas these did not occur when balls were offered as enrichment material.
14	A choice test showed that anYmals highly preferred ropes over balls and balls over chains.

To assess welfare comprehensively, it is necessary to identify all the welfare needs of the animal species of interest (Fig. 1). For the imaginary anYmal, we limited the number of welfare needs to just three needs, namely social contact (as covered by the attribute of group size), exploration (of enrichment material), and movement (space allowance). To calculate welfare scores for anYmals in intensive, semi-intensive, and extensive housing systems, we must identify in these scientific statements welfare-relevant variables of these systems, so-called attributes (with attribute levels that specify housing-system characteristics) that are linked to the needs. In semantic modelling, the term attribute level is used to denote a welfare-relevant property of housing systems in the assessment domain (e.g. the amount of space per pen or number of animals in the group). Each attribute has two (or more) attribute levels, where the levels are determined by a formal decomposition of scientific statements reporting scientific findings in the literature. The levels of an attribute are determined by what is known about that welfare variable scientifically (e.g. about group size), and, together, the attribute levels must also cover the whole assessment domain (i.e. for every housing system that can be assessed with the model, at least one attribute level is true for each attribute in the model; Bracke et al., 2002a). For example, if the attribute of enrichment material has just two levels, e.g. (i) chain and (ii) ball, this means (a) that all housing systems covered by the model can be described as either providing a chain or providing a ball and (b) that there is at least one scientific statement showing a (significant) difference using some welfare measure (e.g. aggression or preference) between the provision of a ball and a chain. In the anYmal example, statements 12–14 provide the scientific basis for chain and ball as levels of the attribute of enrichment material (Table 3), and Table 2 shows that, for this attribute, all housing systems (I, S, and E) are covered by these two attribute levels.

### 2.2 Weighting the welfare impact of attribute levels with weighting-category level scores

If we want to know how good a certain housing system for anYmals is, we need to identify the different properties of a housing system and what is known about their impact on anYmal welfare. As mentioned before, in semantic modelling, the welfare impact of the attribute levels describing a housing system is assessed based on scientific information. To this end, scientific statements about attributes of interest are selected from the scientific literature and decomposed to extract the relevant information for the model (see Vonholdt-Wenker et al., 2026, for more details on how to process scientific statements). Scientific statements typically describe the relationship between environment-based welfare input (i.e. attribute levels, dependent variables, experimental treatments) and animal-based welfare output (independent variable, welfare measure). For example, “if animals are exposed to environmental feature E1 compared to E2 then they show a (significant) increase or decrease of welfare measure M, implying that E1 is better or worse than E2 for welfare (other things being equal)” (see Part 2: Vonholdt-Wenker et al., 2026).

In addition to the identification of the various attribute levels and their relative ranking (and attribute level scoring) for welfare, it is important to determine the relative distance between the best and worst level of each attribute. This, in other words, implies that we must determine how important for welfare one attribute is compared to the other attributes in the model. For this, semantic-modelling principles suggest using weighting factors (WFs) of attributes. WFs can be calculated based on weightings, technically called weighting-category level scores (WCatLevScs). WCatLevScs are assigned to attribute levels based on the decomposition of the scientific statements. Thus, we use the scientific statements about anYmal welfare to determine the positive and negative effects of the attribute levels in the anYmal welfare model (Fig. 1). Given that scientific studies report a large array of welfare measures, we classify those welfare measures for modelling. Here, so-called weighting categories (WCats) are used to classify positive and negative welfare measures broadly related to the different scientific disciplines involved in the study of animal welfare (Table 1). The 12 WCats used in the ANyWEL framework were adopted from the SOWEL model (Bracke et al., 2002a). Together they cover all existing welfare measures from the various disciplines used to assess animal welfare, i.e. ethology, (evolutionary) biology, (stress-related) physiology, and veterinary science. There are nine negative WCats (i.e. pain, illness, survival, fitness, (activation of the) hypothalamic–pituitary–adrenocortical (HPA) axis, sympathetic adrenal medullary (SAM), aggression, abnormal behaviour, and frustration and avoidance) and three positive WCats (i.e. natural behaviour, preference, and demand) (Bracke et al., 2002b). The welfare impact of, especially, the best and worst levels of each attribute are determined using WCatLevScs (Table 1; Fig. 1; see also Vonholdt-Wenker et al., 2026). Each WCat has its own range of assignable WCatLevScs based on the potential impact on biological functioning of the species for each WCat. The WCats indicating positive welfare have positive WCatLevScs, while the negative WCats have negative scores. According to the weighting methodology proposed by Bracke et al. (2002b), WCatLevScs are assigned based on the magnitude (i.e. intensity, duration, and incidence) of the welfare effect of one attribute level compared to another level of that attribute (e.g. 1 $m^{2}$ versus 5 $m^{2}$ per animal). The magnitude of the effect on biological functioning differs between WCats. The 12 above-mentioned WCats have, therefore, been classified into relatively low-impact and high-impact WCats. Overall, the positive WCatLevScs +1 , +3 , and +5 are used for the higher-impact WCat, i.e. demand. Similarly, WCatLevScs of -1 , -3 , and -5 are used for the relatively higher-impact negative WCats of pain, illness, survival, HPA, and frustration and avoidance. For the lower-impact WCats, the WCatLevScs of +1 , +2 , and +3 are used for the positive WCats of natural behaviour and preference, and negative WCatLevScs of -1 , -2 , and -3 are used for the negative WCats of fitness, SAM, aggression, and abnormal behaviour (Bracke et al., 2002b). In order to standardise the process of assigning WCatLevScs in ANyWEL, guidelines for decomposing statements and assigning WCatLevScs have been formulated in another publication (Vonholdt-Wenker et al., 2026). Modellers rely on the semantics of terms used in the scientific statements. For example, words like “highly”, “substantial”, “chronic”, and “essential” are indicative of elevated welfare impacts and thus warrant the assignment of higher WCatLevScs (Vonholdt-Wenker et al., 2026). For the anYmal example, this means that, because the scientific statement that anYmals highly prefer ropes over balls and balls over chains uses the word “highly”, a WCatLevSc of +3 is assigned for the WCat of preference to the attribute level of rope compared to the level of chain, and a preference-related WCatLevSc of +1 is assigned for the attribute level of ball as this is the second preferred object. In addition, the attribute level of chain receives a WCatLevSc of -1 for the WCat of frustration and avoidance as this is by far the least preferred object that is then considered to be avoided when the other objects (either rope or ball) are offered as well (Table 4). Note that it is not the number of words that add weight but the semantic meaning of the used term (i.e. indicating the magnitude of the welfare effect (see also Vonholdt-Wenker et al., 2026)). Semantic modelling uses the biological meaning of the underlying scientific information based on the common conceptual framework for science-based animal welfare assessment (Anonymous, 2001).

**Table 4:** Example with a limited selection of three statements (out of 14 shown in Table 3) about anYmal welfare to illustrate how information from a scientific statement is used to assign weighting categories (WCats) and weighting-category level scores (WCatLevScs) for different types of welfare measures related to the attribute levels referred to in the statement (see Vonholdt-Wenker et al., 2026, for more details on statement decomposition). StatID: statement identification number; ALRank: attribute level rank; Idem: same content as the cell above.

StatID	Scientific statement	Attribute name	Attribute level	ALRank	WCat	Type of welfare measure	WCat LevSc	Welfare need
1	A choice test revealed that anYmals enter more-crowded pens (stocked with 1 $m^{2}\;{\mathrm{anYmal}}^{-1}$ ) much more often than less crowed pens (stocked with $>1m^{2}\;{\mathrm{anYmal}}^{-1}$ ).	Space allowance	1 $m^{2}$	1	Preference	Choice test	+3	Movement
1	Idem	Idem	$>1\;m^{2}$	2	Frustration and avoidance	Idem	-1	Idem
8	Groups of 3 anYmals often fight heavily, resulting in high mortality rates, compared to groups of 8 or 15 anYmals.	Group size	3 anYmals	2	Pain	Fights	-5	Social contact
8	Idem	Idem	8 anYmals	1	Idem	Idem	-1	Idem
8	Idem	Idem	15 anYmals	1	Idem	Idem	-1	Idem
8	Idem	Group size	3 anYmals	2	Aggression	Fights	-3	Idem
8	Idem	Idem	8 anYmals	1	Idem	Idem	-1	Idem
8	Idem	Idem	15 anYmals	1	Idem	Idem	-1	Idem
8	Idem	Group size	3 anYmals	2	Survival	Mortality rate	-3	Idem
8	Idem	Idem	8 anYmals	1	Idem	Idem	-1	Idem
8	Idem	Idem	8 anYmals	1	Idem	Idem	-1	Idem
14	A choice test showed that anYmals highly preferred ropes over balls and balls over chains.	Enrichment material	Ropes	1	Preference	Choice test	+3	Exploration
14	Idem	Idem	Balls	2	Idem	Idem	+1	Idem
14	Idem	Idem	Chains	3	Frustration and avoidance	Choice test	-1	Idem

When decomposing scientific statements each comparison between two attribute levels is weighted by assigning the WCatLevScs and WCat for every relevant welfare measure. This procedure is needed for welfare weighting between attributes (i.e. obtaining the WF of each attribute in the model), as well as for welfare ranking and subsequent scoring of the levels within attributes (i.e. obtaining ALevScs).

Levels of an attribute can be ranked from best to worst based on (the decomposition of) the scientific statements that report welfare-relevant findings about the levels of that attribute (Fig. 1 and Table 5). An imaginary scientific study revealed that anYmals prefer balls over chains (e.g. statement 12: “The duration of time spent playing with balls was higher than the time spent playing with chains.”, Table 3). This means we can rank the levels of the attribute of enrichment material (balls are preferred over chains). Just based on statement 12 alone, the attribute level of ball would then be the best level (i.e. best for the welfare of anYmals) (rank 1), and chain would be the worst level (rank 2). Scientists also reported (in this imaginary case) that anYmals prefer ropes over balls (statement 14: “A choice test showed that anYmals highly preferred ropes over balls and balls over chains”, Tables 3 and 4). Statement 14 identifies a third attribute level of rope, for which the now available scientific evidence warrants classifying rope as the best level (rank 1) followed by ball as the intermediate level (rank 2) and chain as the worst level (rank 3; Table 5). In the end, a housing system can receive the maximum overall welfare score (OWSc) of 1 when the best level of each attribute applies in that system. This implies that all welfare needs are maximally satisfied (in as far as this is supported by the available scientific evidence). This may be a theoretical case because, normally, existing systems have a combination of good and less good welfare properties, though the proportion differs between systems (Bracke et al., 2001).

**Table 5:** Example of ranking attribute levels from best to worst. ALevSc: attribute level score.

Attribute	Attribute level	Order	Rank	ALevSc^a^
Enrichment material	Rope	Best	1	1
	Ball	Intermediate	2	0.5
	Chain	Worst	3	0
Space allowance	1 $m^{2}\;{\mathrm{anYmal}}^{-1}$	Best	1	1
	5 $m^{2}\;{\mathrm{anYmal}}^{-1}$	Worst	2	1
Group size	15 anYmals	Best	1	0.5
	8 anYmals	Intermediate	2	0
	3 anYmals	Worst	3	0

^a^ The attribute level scores (ALevScs) are assigned to each attribute level on a scale between 0 (worst level) and 1 (best level) proportionally to their welfare rank (Bracke et al., 2002b; De Mol et al., 2006); for more details, see also Sect. 3 and Eq. (1a).

After determining the rank of each level of an attribute and assigning the WCats and WCatLevScs based on the scientific statements, the model can calculate the weighting factor (WF) for each attribute. In the next section (Sect. 3), we will explain the various steps needed to calculate the overall welfare score (OWSc) for each housing system using the anYmal example.

## 3 Model calculation steps to produce the overall welfare score for each housing system

Before we illustrate how to calculate welfare for the imaginary anYmal species, we refer the reader to Table 2, describing the three housing systems (intensive, I; semi-intensive, S; and extensive, E). For these housing systems, we calculate an overall welfare score (OWSc) based on anYmal welfare science. The calculation steps are based on the principles formulated previously for the SOWEL model (Bracke et al., 2002a, b). As illustrated in Fig. 1, the model contains attributes with levels, attribute level scores (ALevScs), and weighting factors (WFs), such that OWScs can be calculated for each housing system. To this end, the model user needs to specify for each attribute which attribute level is applicable to the housing system of interest. Since all attribute levels have been assigned attribute level scores (ALevScs, based on their welfare ranks) and since weighting factors (WFs) have been calculated for attributes in the modelling process, the model allows the calculation of OWScs for the housing systems. The following formulas are used to calculate the OWScs:

*Attribute level scores (ALevScs).* The ALevScs are calculated for each attribute level on a scale between 0 (worst level) and 1 (best level) proportionally to their rank (Bracke et al., 2002b; De Mol et al., 2006) (see Eq. 1a and Table 5).

1a $${\text{ALevSc}}_{ij}=\frac{{\text{NL}}_{i}-{\text{RL}}_{ij}}{{\text{NL}}_{i}-1}$$

In the above, ${\text{ALevSc}}_{ij}$ represents the attribute level score of attribute level j of attribute i . The total number of levels within attribute i is denoted by ${\text{NL}}_{i}$ . The rank of attribute level j of attribute i is denoted as ${\text{RL}}_{ij}$ (see Bracke et al., 2002b). The attribute of enrichment material may serve as an example. This attribute has three levels ranked from best to worst: rope, ball, and chain. Here, Eq. (1a) generates the ALevScs of 1, 0.5, and 0, respectively, as shown in Table 5, using Eq. (1b–d):

1b $$\text{ALevSc (Rope)}=\frac{3-1}{3-1}=1,$$

1c $$\text{ALevSc (Ball)}=\frac{3-2}{3-1}=0.5,$$

1d $$\text{ALevSc (Chain)}=\frac{3-3}{3-1}=0.$$

The general rule is that intermediate levels (between best, 1, and worst, 0) are given intermediate scores proportional to the number of levels on the scale (0–1). For an attribute with one intermediate level (and three levels in total), the intermediate level gets an ALevSc of 0.5, whereas two intermediate levels get ALevScs of 0.33 and 0.67, respectively, depending on their rank, etc. Note that this equidistance principle between levels is a rule of thumb. The relative distance between any two levels of an attribute containing more than two levels can, in principle, be derived in a way that is similar to the procedure that is used to derive WFs (which reflect the relative distance between the best and worst levels of each attribute). Note, furthermore, that since rope is not actually provided in any of the three existing housing systems (I, S, and E) of anYmals, this best level (rope) of the attribute of enrichment material should be eliminated from the model when the model is designed to only apply to “existing systems” (i.e. existing in the fictitious example). In that case, ball would be the best level and, hence, would have an ALevSc of 1 instead of 0.5. This illustrates how the definition of the assessment domain may affect the model's properties (attribute levels, ALevScs, and WFs of the attribute of enrichment material).

*Weighting factors (WFs).* The WF denotes the relative importance of an attribute compared to the other attributes in the model. The greater the WF, the more the attribute is contributing to overall welfare (which is calculated as a weighted average of the ALevScs). The WF can be calculated technically as the difference between the sum of the welfare benefits (minus the welfare disadvantages, if there are any) of the best level and the sum of the welfare disadvantages (minus the benefits) of the worst level of the attribute. This is represented by the difference between the sum of the maximum (highest) WCatLevScs of the best level minus the minimum (i.e. lowest) values of the negative WCatLevScs of the worst level of the attribute. In addition to the highest and lowest WCatLevScs per WCat, some additional weight is generated by the number of different types of welfare measures (called types) per WCat. This reflects the idea that different welfare measures add weight, especially across disciplines (rather than within WCats), and that, as such, repeated confirmation using exactly the same welfare measures does not add any weight. Scientific statements may load on one attribute level (compared to another level) with different types of welfare measures within the same WCat, e.g. different types of disease, disease symptoms, or immunological measures all classified in the WCat of illness. For instance, in the example statement 10 (“AnYmals kept in groups of 3 showed a much higher number of life-threatening diseases, such as bacterial and viral respiratory infections due to a low immune response, than in groups of more than 10 anYmals”, Table 3). Bacterial infections and viral respiratory infections are two different types of welfare-relevant measures of the WCat of illness. The inclusion of such different types of welfare measures about the same (best or worst) attribute level should affect the WF of the attribute (and, hence, the overall welfare score, OWSc) because, other things being equal, such additional information (i.e. two types rather than one) should make that attribute a little more important. Therefore, the weightings of the best and worst level can be calculated from the highest and lowest WCatLevScs per WCat, respectively, and from the number of different types (of welfare measures) per WCat assigned to the best and worst attribute levels (Fig. 1). Consequently, the weighting factor (WF) can be calculated as follows (Eq. 2a):

2a $$\begin{array}{rl} {\text{WF}}_{i}= & (\sum_{\text{WCat}}\text{Max}\;({\text{WCatLevSc}}_{\text{WCat}})\\ & +\sum_{\text{AD.Type}}({\text{Max WCatLevSc}}_{\text{WCat}}\times 0.2{)}_{\text{Type}}{)}_{\text{ALbest}}\\ & -(\sum_{\text{WCat}}\text{Min}({\text{WCatLevSc}}_{\text{WCat}})\\ & +\sum_{\text{AD.Type}}({\text{MinWCatLevSc}}_{\text{WCat}}\times 0.2{)}_{\text{Type}}{)}_{\text{ALworst}}. \end{array}$$

Here, i.e. in the ANyWEL framework, ${\text{WF}}_{i}$ represents the absolute weighting factor of attribute i . ${\text{WF}}_{i}$ is the difference (i.e. the middle min (–) sign in the formula) between two terms (between the larger round brackets in Eq. 2a). The first term (a) is the sum (plus sign) of the highest weighting-category level score (WCatLevSc) of each weighting category (WCat) of the best attribute level ( ${\text{AL}}_{\text{best}}$ ) supplemented with one-fifth (0.2 times) of the highest WCatLevScs of any additional and distinct type of welfare measure for each WCat related to the best attribute level ( ${\text{AL}}_{\text{best}}$ ). In Formula 2a, the term AD type stands for each additional (A) and distinct (D) type of welfare measure. The second term (b) is the sum (second plus sign) of the lowest (minimum) WCatLevSc of each WCat associated with the worst attribute level ( ${\text{AL}}_{\text{worst}}$ ) and 0.2 times the most negative (min) WCatLevSc of each additional and distinct type associated with ${\text{AL}}_{\text{worst}}$ . This differs from the SOWEL model, where, for every distinct type within a WCat, 0.2 was added to the WF regardless of the WCatLevSc associated with that type (of welfare measure).

The new ANyWEL rule is a clear improvement. This can be illustrated by the attribute of enrichment material (for anYmals) with its ${\text{AL}}_{\text{best}}$ rope and ${\text{AL}}_{\text{worst}}$ chain. Here, the outcomes of a choice test (statement 14) imply for ${\text{AL}}_{\text{best}}$ rope a WCatLevSc of +3 for the WCat of preference and a WCatLevSc of -1 for the WCat of frustration and avoidance for ${\text{AL}}_{\text{worst}}$ chain (Table 5). In statement 14, there were no (i.e. 0) additional types described for ${\text{AL}}_{\text{best}}$ rope. So, there is no added weight derived from any additional and distinct types of welfare measure. Therefore the sum of WCatLevScs for this ${\text{AL}}_{\text{best}}$ is only the + 3 for preference. For ${\text{AL}}_{\text{worst}}$ chain, there are two types of welfare measure belonging to the WCat of frustration and avoidance as statement 12 (Table 3) reported that anYmals preferred balls over chains based on the time spent playing (i.e. voluntary interaction with the material). Furthermore, mouth and teeth injuries were reported in anYmals when chains were offered (statement 13, Table 3), indicating two more types for the WCat of pain, both of which were scored with a WCatLevSc of - 1. Hence, the sum of WCatLevScs for ${\text{AL}}_{\text{worst}}$ chain is calculated as $\left(-1_{\text{Frustration}})+(-0.01_{\text{Frustration}}\times 0.2)+(-1_{\text{Pain}})+(-1_{\text{Pain}}\times 0.2\right)$ (see Sect. 4 and Vonholdt-Wenker et al., 2026, for details about assigning a ±0.01 WCatLevSc). Now, the WF for the attribute of enrichment material can be calculated as follows (Eq. 2b):

2b $$\begin{array}{rl} & {\text{WF}}_{\left(\text{Enrichment}\right)}=((+3_{\text{Preference}})+(0\times 0.2){)}_{\text{ALBest(Rope)}}\\ & \;-((-1_{\text{Frustration(choice test)}}\\ & \;+(0.2\times -0.01_{\text{Frustration (time spent playing)}}))\\ & \;+(-1_{\text{Pain(teeth injury)}}+(0.2\times \\ & \;-1_{\text{Pain(mouth injury)}})){)}_{\text{ALWorst(Chain)}}\\ & =+3_{\text{ALBest(Rope)}}-(-2.2_{\text{ALWorst(Chain)}})=5.2. \end{array}$$

The WF of 5.2 for the attribute of enrichment material means that the best level of that attribute (rope) with an ALevSc of 1 is counted 5.2 times in the calculation of the OWSc.

Additional examples of WF calculations can be found in Supplement S1. Generally, the meaning of a WF is that the higher the WF of an attribute is the more important the attribute is for welfare relative to the other attributes in the model. After the absolute WF for each attribute has been calculated, we sum up all the WFs of all attributes in the model ( ∑WF ). As can be seen from Table S1A1 in the Supplement S1, the attribute of group size has a very high WF. Table 3 shows the imaginary scientific statements on which this is based. Table 4 shows the decomposition of statement 8, reporting often heavy fighting and elevated mortality in groups of three compared to in groups of five or eight. This is linked in Table 4 to three WCats, namely aggression, pain, and fitness, because that is what (we believe) this finding means for welfare (and this is an important fact to take into account). The associated WCatLevScs then generate a relatively higher WF for this attribute (group size) relative to the other attributes (space allowance and enrichment material).

For our anYmals in the assessment domain, the weighting factor sum ( ∑WF ) is 32.92 (see Table S2B1 in the Supplement).

*Weighted attribute level scores (WALevScs).* The next step in model construction is to calculate for each attribute level its weighted attribute level score (WALevSc). This is done by multiplying the ALevSc (Eq. 1a) of each level of attribute i with the absolute ${\text{WF}}_{i}$ , i.e. the weighting factor of attribute i (Eq. 3a).

3a $$\text{WALevSc}=\text{ALevSc}\times {\text{WF}}_{i}$$

For example, to calculate the WALevSc of the attribute level of ball of the attribute of enrichment material, the WF of this attribute (i.e. 5.2) is multiplied by the respective ALevSc (i.e. 0.5 for Ball) (see Eq. 3b):

3b $${\text{WALevSc(Enrichment}}_{\text{Ball}})=0.5\times 5.2=2.6.$$

*Overall welfare score (OWSc) per housing system.* OWScs can be calculated for each housing system in the model's assessment domain by identifying exactly one attribute level for each attribute in the model (that best describes the housing system), identifying its WALevSc, adding all WALevScs up ( ∑WALevSc ), and dividing them by $\sum {\text{WF}}_{i}$ (Eq. 4a). In this way OWSc is calculated as a weighted average of the ALevScs. The OWSc of a housing system (HS) can range between 0 and 1, where 1 represents the best possible housing condition and 0 represents the worst according to the model.

4a $${\text{OWSc}}_{\text{HS}}=\frac{\sum {\text{WALevSc}}_{\text{HS}}}{\sum \text{WF}}$$

**Figure 2:**
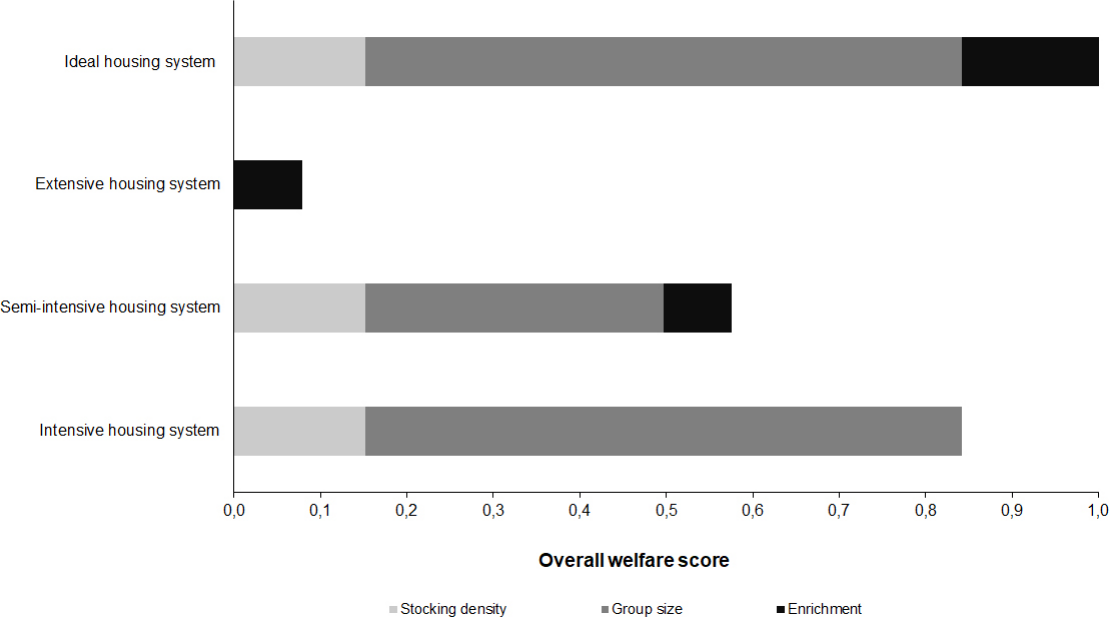
The overall welfare scores for three different housing systems (intensive, semi-intensive, and extensive) and an ideal housing system for the fictitious species anYmal. The different colours reflect the respective shares of the three welfare-relevant attributes in the total overall welfare score for each housing system. In case the worst level of an attribute is present in a housing system, it does not contribute to welfare and so is not shown in the figure (e.g. space allowance and group size for the extensive system). The length of the three bars of the ideal system represents the weighting factors (WFs) of the three attributes in the model (with group size being by far the most important due to the scientific statements presented in Table 3 about this species).

For anYmals, we calculated an OWSc of 0.84 for the intensive housing system, 0.58 for semi-intensive housing, and 0.08 for the extensive system (Eq. 4b–d; S2 in the Supplement). Alternatively, each WALevSc (of all levels of all model attributes) can be divided by $\sum {\text{WF}}_{i}$ separately, even before we calculate OWScs. These could be called normalised WALevScs. Adding up the normalised WALevScs for all of the attributes of a housing system adds up to the OWSc (as e.g. shown in Fig. 2, where the ideal system shows the maximum contribution of the best level of each attribute to overall welfare).

4b $${\text{OWSc}}_{\left(\text{Intensive}\right)}=\frac{27.7}{32.9}=0.84$$

4c $${\text{OWSc}}_{\left(\text{Semi-intensive}\right)}=\frac{19.0}{32.9}=0.58$$

4d $${\text{OWSc}}_{\left(\text{Extensive}\right)}=\frac{2.6}{32.9}=0.08$$

Based on these calculations and the underlying scientific statements about the welfare of anYmals, the intensive housing system is a more welfare-friendly system for anYmals in that it better meets their welfare needs compared to the semi-intensive and extensive systems (Fig. 2). This counter-intuitive result is the direct consequence of the fictitious scientific knowledge postulated about these imaginary anYmals (Table 3). This also highlights that modelling approaches can reveal patterns that are counter-intuitive to experts and may thus help in unravelling novel directions for improving the welfare of farm animals (see e.g. Ursinus et al., 2009; Bracke and Spoolder, 2011).

Overall, the anYmal example demonstrated how the various model components (i.e. scientific statements, needs, attributes, attribute levels, attribute level scores, weighting categories, and weighting-category level scores) and underlying procedures and calculations within the semantic ANyWEL model framework allow the construction of welfare models based on scientific information for different species of farm animals. With these models, users may calculate overall welfare scores for housing systems in the model's assessment domain. During the development of the ANyWEL model framework, the calculation rules were improved. In particular, the rules for weighing the types of welfare measurement and assigning WCatLevScs were adjusted to improve the overall welfare assessments. These refinements will be explained in the next section (Sect. 4).

## 4 Model refinements within the ANyWEL model framework

### 4.1 Types of welfare measures

As mentioned earlier, types characterise the specific type of welfare measure within a WCat. Types thus enable the differentiation of the measured welfare effects within a WCat (Bracke et al., 2002b). Including types adds a bit more weight to the WF of an attribute on top of the fact that the magnitude of the WF is mainly determined by the difference between the sums of the highest (most positive and negative) WCatLevScs of each WCat (for the best and worst level of the attribute, respectively). In order to be able to take the WCatLevSc of different types within a WCat into account, we refined the calculation rule for types as originally described for the SOWEL model developed by Bracke et al. (2002b). By doing so, the integration of the welfare impacts (e.g. bacterial infection and viral infection within the WCat of illness) should be more accurate, i.e. more related to the actual weight (WCatLevSc) each distinct type loads onto the WF compared to the calculation rule used in the SOWEL model. In SOWEL types of welfare measures load onto the attribute's WF by adding one-fifth (0.2 times) the total number of different types per WCat to the WF, i.e. the total number of different types was multiplied by 0.2 (see Supplement S1 and Bracke et al., 2002b). We consider this procedure to be suboptimal as SOWEL thus only looks at the total number of different types within a WCat irrespective of the actual (higher or lower) WCatLevScs that were related to each distinct type of welfare measure. The new calculation rule prescribes a multiplication of the highest and lowest assigned WCatLevSc for each different type with 0.2 (highest for the positive WCats and lowest for the negative WCats; see Eq. 2a and Supplement S1).This new rule differs from the original rule proposed in SOWEL in that multiple measures (types) within a WCat now generate a higher WF (compared to the original rule), especially when these types are associated with relatively high WCatLevScs (i.e. higher than 1 so especially scores of ±3 or ±5 ). Supplement S1 shows example calculations using both the original SOWEL and the new ANyWEL calculation rule.

### 4.2 Weighting-category level scores (WCatLevScs)

Previous models worked with WCatLevScs on scales of either ±1 –3 or ±1 –5 (depending on the corresponding WCat) (Bracke et al., 2002b; De Mol et al., 2006; Ursinus et al., 2009). However, in the development of the ANyWEL framework, we suggest improving the weighing process (compared to the previous models including SOWEL): we defined several additional levels to assign weightings (i.e. new WCatLevScs) in the generic ANyWEL model. By these means, we expect that the OWSc in ANyWEL is calculated more accurately.

*Refined WCatLevScs.* The first refinement of the WCatLevScs in ANyWEL was to increase the range of WCatLevSc for the WCat of frustration and avoidance. Bracke et al. (2002b) considered demand to be the strongest positive WCat and therefore chose the positive scoring scale from +1 to +5 . Demand shows that an animal is willing to work to obtain a commodity. The negative complement of positive demand (i.e. wanting something) is the WCat of frustration and avoidance, which may be taken to include a more or less strong motivation to avoid, including, for example, a strong “demand” to avoid a painful or frightening stimulus (Kirkden and Pajor, 2006). Given that positive demand received relatively high-impact WCatLevScs (1–5), it seems reasonable to propose a similar scoring range for negative demand, i.e. for the WCat of frustration and avoidance. The negative emotions related to frustration and avoidance may even be stronger than those for positive demand (with the 1–5 scale in Bracke et al. 2002b). Thus, we adjusted the scale of WCatLevSc for frustration and avoidance from -1 to -3 , as in the SOWEL model, to a scale from -1 to -5 in ANyWEL. Note also that the low end of the scale (i.e. 1 and -1 ) is now, i.e. as of the ANyWEL model framework, more explicitly postulated as being represented by a significant finding (of a measure indicating a positive or negative welfare effect, respectively). In the original SOWEL model this was more or less tacitly assumed but not explicitly stated. Another tacit assumption in SOWEL was the fact that the actual scale of WCatLevScs ranged between 0 and ±3 or 5 rather than starting from ±1 . This starting point at 0 rather than ±1 is now made more explicit (see also below).

*Expert opinions.* Furthermore, Bracke et al. (2002b) reported that, due to a lack of available publications, difficulties can arise in assessing the welfare impact of attributes. This is especially the case when specific properties (attribute levels) of rather new and innovative housing systems cannot be fully evaluated due to a lack of published scientific studies. Therefore, as a second refinement, we developed intermediate WCatLevScs for cases in which sources other than the scientific literature are considered (e.g. expert or panel assessments). In SOWEL, gaps in knowledge were filled by the modeller themself and were not further investigated. This is because the inserted knowledge gaps were considered to have expert consensus (like the fact that animals without food or water will die, even when no studies collected in the database experimentally reported this). Building on this principle, we allow the inclusion of a wider range of (less confident) expert opinions in the ANyWEL framework. Since expert opinions are a source of information with a lower reliability than proper and peer-reviewed scientific statements, we propose three additional WCatLevScs (on the existing scales of ±1 –3 and ±1 –5) that have a lower weighting than the previous lowest score ( ±1 ) to be used for expert, committee, or panel assessments, i.e. ±0.10 , 0.25, and 0.40 (for both the positive and negative WCats; see below). By using these additional WCatLevScs, expert assessments can be included in the ANyWEL model to supplement the statements from the scientific literature. Thus, like the WCatLevScs used for published statements, WCatLevScs for expert-elicited statements can be assigned depending on the duration, incidence, and intensity that experts expect for certain welfare impacts of an attribute. Relatively small effects receive a WCatLevSc of ±0.10 (i.e. a 10-fold-reduced WCatLevSc instead of the original WCatLevSc of ±1 ). So, as it were, 10 expert opinions on a given subject would count as much as 1 statistically significant experimental finding in the literature. Medium effects receive a WCatLevSc of ±0.25 , and large effects can be given a score of ±0.40 (see Vonholdt-Wenker et al., 2026). Nevertheless, the gold standard for data acquisition should be to include scientific statements from peer-reviewed publications as much as possible to preserve the objective and scientific basis of the model.

Consequently, new studies could be published about attribute levels that were previously assessed by experts. For this reason we implemented a new WCatLevSc of 0 in the ANyWEL model. This WCatLevSc can, in particular, be used to replace expert assessment by literature statements. In case there is both an expert opinion and a published statement regarding the same attribute level (and the same type of welfare measure), it is recommended to use only the published statement to do the calculations due to the higher reliability. At the same time, the corresponding expert opinion should be set to 0 (which means no weight is assigned anymore). This allows transparency by, on the one hand, leaving the expert assessment in the statement database but not taking it into account anymore for the model calculations and, at the same time, archiving it, which enables a subsequent improvement of the dataset. Thus, no statements get lost in the database, and no distortions are generated (by additionally considered expert opinion) by “double” counting in the calculation.

*Reduced reliability score.* Similarly to the WCatLevSc for expert assessments with a lower reliability, we created a third refinement for the WCatLevSc for published statements reporting results that were not statistically significant ( P<0.05 ). Tendencies ( P<0.10 ) could point towards potentially interesting findings that suggest some likelihood of becoming significant with larger sample sizes or repeated testing and meta-analysis. Therefore, tendencies (or other relatively weak statements) can be included in the ANyWEL model using a WCatLevSc of ±0.5 . This score can be used for all WCats. By including this score, more literature statements can be included in the model and contribute to a more robust welfare assessment. Alternatively, this score can also be used if modellers consider certain studies to be of rather “poor” quality despite the statistically significant results. Including tendencies may also reduce publication bias, which could occur if only significant findings were considered. However, some publication bias will remain as some welfare aspects are much more difficult to study than others. Accordingly, the likelihood of retrieving reported significant findings is higher. Semantic modelling cannot solve this. What it can do is help identify such phenomena and gaps in knowledge.

*Opposing weighting-category level scores.* Statements always contain a comparison between at least two levels of an attribute, e.g. as in statement 12, which states “The duration of time spent playing with balls was higher than the time spent playing with chains”. This statement says that the level of ball of the attribute of enrichment material was preferred over chain (as measured by the time spent playing). This statement can be decomposed by assigning a positive WCat (i.e. preference) for ball compared to chain. In the statement, ball is the best level, and chain is the worst attribute level. As chain would be the worst attribute level until other statements allowing for higher WCatLevScs have been found, we introduce opposing scores, with a near-0 score. For this we propose a WCatLevSc of ±0.01 (see Supplement S1). With this score we can assign some value to the opposite attribute level (compared to the one receiving the main WCatLevSc). This is done to allow the calculation of the WF even when no other WCatLevScs are assigned for this attribute (see Vonholdt-Wenker et al., 2026).

## 5 Discussion

The objectives of this paper were (i) to explain the general process of semantic modelling and guide the reader through the improved semantic ANyWEL framework with the use of a fictitious animal species called anYmal and (ii) to reflect on the strengths and potential limitations of this model. We have shown how the ANyWEL framework can calculate overall welfare scores (OWScs) for different housing systems in a standardised and formalised way across species using an exemplary anYmal and postulated scientific knowledge to show how the scientific information about the species determines the outcome (rather than what may be expected based on casual generalisation from existing species). Below, we discuss refinements that were implemented in the ANyWEL model compared to previous semantic models (Bracke et al., 2002b) and evaluate the strengths and limitations of our semantic-model framework to indicate why ANyWEL is a major step forward in semantic modelling. In essence, the ANyWEL framework was developed based on the initial concept of the SOWEL model (Bracke et al., 2002b). However, several notable refinements were made by us to enhance the accuracy of the welfare assessment, i.e. the calculation of OWScs.

The ANyWEL framework described in this paper was designed as part of the InKalkTier project (https://www.ktbl.inkalktier.de, last access: 3 July 2026) to provide guidance to new modellers. For the first time, statistically significant welfare findings were presented as the basic unit of overall welfare calculations (by ranking of attribute levels leading to attribute level scores and through the assignment of WCatLevScs). Moreover, the ANyWEL model framework can be used to assess the welfare of any farm animal species. Previous semantic welfare models, such as FOWEL (De Mol et al., 2006) for laying hens, SWIM 1.0 and SWIM 2.0 (Stien et al., 2013; Pettersen et al., 2014) for Atlantic salmon, and COWEL (Ursinus et al., 2009) for dairy cows, had been developed, with modifications, for specific production directions. The ANyWEL approach now allows modellers to assess overall animal welfare in various production directions within the same framework. This is expected to further enhance modelling efficiency and standardisation. Moreover, compared to previous models, we believe ANyWEL is better suited to assess innovative housing systems that lack scientific studies. For this, refined rules were suggested for the incorporation of expert opinions. By including the refined WCatLevScs for weaker statements expressing opinions and tendencies, the ANyWEL model should be able to include more knowledge and calculate animal welfare more accurately. Another advantage of this innovation is the possibility to further study the similarities and differences between welfare assessments based on the published scientific literature and based on “mere” (expert) opinions. This could potentially help bridge the gap between science and society. A major strength of the framework, however, is that it is explicitly and formally based on (the collection and decomposition of) scientific knowledge. A possible weakness associated with this is the existence of knowledge gaps (i.e. gaps in the literature and in what can be retrieved from expert opinion). Such gaps could potentially negatively affect the weighting factors (WFs). As stated above, the WF of an attribute represents how much this attribute contributes to the welfare of an animal (i.e. how important the resource is to the animal and its welfare needs). However, the WF can only be calculated through collecting published or elicited scientific statements, resulting in ranked attribute levels and WCatLevScs (Bracke et al., 2002b, 2008a; Ursinus et al., 2009). Some attributes or interactions between attributes may affect welfare without having been duly considered in scientific studies (e.g. water trough design for dairy cattle seems to be understudied; Pinheiro Machado Filho et al., 2004). The ANyWEL framework can handle these issues via the adjusted WCatLevScs for expert opinions. Expert opinions could help fill these publication gaps and thereby reduce the risk of underestimating such welfare-relevant attributes (provided there is sufficiently reliable expert opinion to justify them). However, remaining knowledge gaps that cannot even be solved with expert opinions remain an issue for science to solve rather than something to solve in semantic modelling as such. Semantic modelling can help to identify knowledge gaps, but it is up to science to close these gaps. Once that has been done, existing models can be updated to accommodate the state of the art. The users of semantic models should be aware that attributes may vary in terms of the number and quality of the scientific (or expert-opinion) statements they are based on. This can be mitigated by the transparency provided by the model, as well as by the fact that limited scientific evidence will generally result in lower WFs. Perhaps we can briefly illustrate this: in our imaginary example we referred to enrichment materials like chains and balls. Within the field of pig welfare science, such enrichment materials are now increasingly regarded as (very) insufficient, perhaps not even worthy of being called enrichment material. For such reasons, it is important that modellers conducting the literature searches have a basic understanding of welfare science generally. New studies showing that, for example, straw and peat are much more effective in activating exploratory behaviour in pigs (e.g. Kauselmann et al., 2021) should, however, fairly “automatically” (i.e. when our semantic-modelling guidelines are adhered to) lead to chains and balls acquiring much lower attribute level scores and, hence, contributing less to overall welfare, thus establishing the view that these materials are hardly enriching for the pigs.

To the superficial reader it may seem that the number of scientific statements positively correlates with the weighting factors. However, it is important to note that the WF represents not the number of statements but their scientific meaning: only the maximum and minimum WCatLevScs within a WCat are used in the calculation of the WFs, and in SOWEL only the distinctly different types of welfare measure generate (a little bit of) extra weight. In ANyWEL, however, the other WCatLevScs are also factored in, but only when it concerns additional and distinct (types of) welfare measures. Hence, a higher number of scientific statements can result in a higher WF only when different measures or larger effects have been reported (Ursinus et al., 2009; Bracke, 2008).

Another point to note is that the output of the semantic model should be cross-checked with current practices on farms using, for example, an overall welfare assessment with animal- and environment-based welfare indicators (e.g. those compiled in the Welfare Quality^®^ project, Blokhuis et al., 2010). Other ways of validating the model and its attributes are by using sensitivity analysis, a comparison with another set of scientific knowledge (e.g. scientific studies reported in different periods of time, or in even versus uneven years of publication (Bracke et al., 2004a)), on-farm evaluation (Folkedal et al., 2016), and expert opinion (Bracke et al., 2002b, 2007, 2008a). The ANyWEL framework would certainly benefit from further validation, e.g. by a comparison with expert opinions (e.g. on its modelling principles) and application by independent (groups of) researchers. Even without such validation, semantic models can be used tentatively for welfare screening and the design of new welfare-friendly systems (as has, for example, been done with the COWEL model of Ursinus et al., 2009). For some of these applications, such as the design of new systems, assessments at the system level, and integrated sustainability assessments, semantic models may be the only transparent science-based methods available, though external validation remains desirable. For other applications, like on-farm assessments, a comparison (and validation) may be warranted with other instruments like the Welfare Quality^®^ protocol or qualitative behaviour assessment (QBA, e.g. Cooper and Wemelsfelder, 2020), each of which has advantages and disadvantages in terms of validity, reliability, and feasibility. Note that the relationships with some welfare measures that seem to be able to assess overall welfare like judgement bias tests (JBTs, Neuhauser et al., 2023) and telomere length are multifaceted. Such measures can both be used to validate semantic models (and vice versa) and be classified as other welfare measures and, as such, be used to improve semantic models like other newly available welfare measures or studies would (e.g. JBT may be seen as a special kind of preference test (WCat: preference), while telomere length may be classified in the WCat of fitness). A difficulty here is that, for example, a positive JBT (i.e. showing that anYmals in an enriched environment are more optimistic) generally does not (yet) allow a specification of which properties (attribute levels) of the enriched condition result in the optimism measured with the JBT.

The procedure of semantic modelling itself is highly formalised, comprehensive (taking into account all available knowledge), and designed to take the modeller's point of view out of the evaluation, as has been described in other papers (Bracke et al., 2002b, 2008a). Still, a main criticism in semantic modelling remains the underlying subjectivity (Stien et al., 2013). That problem, however, is inherent in any welfare assessment and even in any welfare experiment, as long as we do not have a valid and direct welfare “thermometer”. For the time being, subjective interpretation of information is necessary to assess animal welfare, and, accordingly, no welfare assessment is value free. Semantic modelling is designed to make the best-possible assessment based on all available and reliable information, i.e. on scientific statements collected in a database. In semantic modelling, the modellers decide which WCat and which WCatLevSc they assign to which attribute level when decomposing a scientific statement. In applying a semantic model, the users must also decide which welfare-relevant attribute levels apply in the housing systems they want to assess. We cannot rule out that modellers and users can make mistakes. Semantic modelling, however, also allows for making all decisions transparent, such that errors can, in principle, be identified, discussed, and corrected. In case of ambivalence, it is possible to run alternative model calculations (e.g. with or without WFs) and settle disputes. For example, sensitivity analysis of existing models, which usually have more than 30 attributes, showed that OWScs calculated with and without WFs (i.e. with all WFs set at 1) were highly similar in terms of output (i.e. OWScs of benchmark systems). This suggests that disputes about the assignment of WCatLevSc may have a relatively minor impact compared to, for example, the number of attributes identified in the model (Bracke et al., 2002a, b; Ursinus et al., 2009). In semantic modelling, the impact of subjective decisions also decreases as the quantity and quality of the scientific statements collected from peer-reviewed publications and other reliable sources increase (Stien et al., 2013). To further formalise and standardise the semantic-modelling procedure of selecting and decomposing scientific statements and objectively weighting the welfare effect, a guideline to enhance inter-modeller reliability has been developed (Vonholdt-Wenker et al., 2026).

In contrast to existing semantic models, which included housing, as well as management, factors in the assessment domain (Bracke et al., 2002b; De Mol et al., 2006; Ursinus et al., 2009; Stien et al., 2013), the ANyWEL model framework was designed to apply to different species, housing systems, and assessment domains, resulting in a specific welfare output which we labelled “welfare potential”. This was done because, for the InKalkTier project, in the context of which ANyWEL was developed, management factors were not to be included in the welfare assessment (i.e. InKalkTier assumed best management practices) – hence the term welfare potential rather than actual or realised welfare. However, we think that the ANyWEL framework can also be applied to construct models that include suboptimal management practices.

The ANyWEL framework, furthermore, allows the development of models for different life stages of the animals, e.g. for pigs in the farrowing, weaned, growing, and fattening stages. In these cases, for example, the available amount of space per pig changes over time (Chidgey, 2023). When the aim is to make the best-possible welfare assessment throughout their entire lifetime, perhaps even including the transport and slaughter phases, surely future work needs to be done to integrate the output of the different component models. In addition to that, further work needs to clarify and formalise the process of combining attribute levels (e.g. combination of perch height and perch shape for laying hens).

Beyond farm animals and welfare, the ANyWEL model framework may be extended to cover other species, e.g. zoo animals, wild animals, laboratory, and companion animals, and perhaps also other (social) sustainability aspects (which seem mostly centred around human welfare). Thus, the suggestion we make here is that the semantic-modelling approach could also go beyond animal welfare to assess the sustainability, such as in terms of environmental and economic impacts, of livestock farming (Bracke et al., 2023). This may makes sense once we realise that ANyWEL is to apply to any animal and that what matters for sustainability is to allow humans and animals to thrive and experience positive welfare (Rault et al., 2025). The ANyWEL framework remains to be implemented in actual welfare models, but welfare statements about various species of livestock have been extracted using the ANyWEL framework (Benthin et al., 2023). These have been successfully used to develop an AI tool for automated statement extraction (Zhang et al., 2026). This is in line with the idea that the principles underlying semantic modelling are bio-logical, i.e. based on (an understanding of) biology and logic (see the explanation of using if–then rules in Vonholdt-Wenker et al., 2026).

## 6 Conclusion

ANyWEL is a novel framework for assessing the welfare state of different species and production stages of farm animals in relation to housing (and management) based on scientific statements. The framework prescribes how to integrate scientific findings to generate welfare scores to compare and benchmark different systems. The ANyWEL framework can be used to make semantic models to calculate overall welfare scores for existing and new housing systems. This can improve insight into the relative contribution of different aspects (attributes) to overall welfare and the design of new welfare-friendly systems. The greatest advantage of the ANyWEL model framework is its ability to standardise welfare assessment across species. Compared to the original semantic model (SOWEL) on which ANyWEL is based, the accuracy of the modelling framework was improved. To this end, we adjusted the calculation rules (e.g. for types of welfare measures and the assignment of weighting-category level scores). The ANyWEL framework was illustrated using a fictitious animal species called anYmal kept in three different housing systems (intensive, semi-intensive, and extensive). We showed how expected and subjective welfare assessment may be modified once scientific knowledge is considered explicitly following the ANyWEL procedures.

## Supplement

10.5194/aab-69-363-2026-supplement The supplement related to this article is available online at https://doi.org/10.5194/aab-69-363-2026-supplement.

## Data Availability

The data presented in this article can be obtained from the authors upon request.

## References

[bib1.bib1] Alonso ME, González-Montaña JR, Lomillos JM (2020). Consumers' concerns and perceptions of farm animal welfare. Animals.

[bib1.bib2] Anonymous (2001). Scientists' Assessment of the Impact of Housing and Management on Animal Welfare. J Appl Anim Welf Sci.

[bib1.bib3] Benthin J, Vonholdt-Wenker M L, Kauselmann K (2023). Zenodo [Data set].

[bib1.bib4] Blanco-Penedo I, Ouweltjes W, Ofner-Schröck E, Brügemann K, Emanuelson U (2020). Symposium review: Animal welfare in free-walk systems in Europe. J Dairy Sci.

[bib1.bib5] Blokhuis H, Veissier I, Miele M, Jones B (2010). The Welfare Quality^®^ project and beyond: Safeguarding farm animal well-being. Acta Agr Scand A An.

[bib1.bib6] Bracke MBM (2001). Modelling of animal welfare: The development of a decision support system to assess the welfare status of pregnant sows [dissertation].

[bib1.bib7] Bracke MBM (2006). Providing cross-species comparisons of animal welfare with a scientific basis. NJAS.

[bib1.bib8] Bracke MBM (2008). RICHPIG: a semantic model to assess enrichment materials for pigs. Anim Welfare.

[bib1.bib9] Bracke MBM, Spoolder H (2011). Review of wallowing in pigs: implications for animal welfare. Anim Welfare.

[bib1.bib10] Bracke MBM, Spruijt BM, Metz J (1999). Overall animal welfare reviewed. Part 3: Welfare assessment based on needs and supported by expert opinion. Neth J Agr Sci.

[bib1.bib11] Bracke MBM, Metz J, Dijkhuizen AA, Spruijt BM (2001). Development of a decision support system for assessing farm animal welfare in relation to husbandry systems: Strategy and prototype. J Agr Environ Ethic.

[bib1.bib12] Bracke MBM, Metz JHM, Spruijt BM, Schouten WGP (2002). Decision support system for overall welfare assessment in pregnant sows B: validation by expert opinion. J Anim Sci.

[bib1.bib13] Bracke MBM, Spruijt BM, Metz J, Schouten W (2002). Decision support system for overall welfare assessment in pregnant sows A: Model structure and weighting procedure. J Anim Sci.

[bib1.bib14] Bracke MBM, Hulsegge B, Keeling L, Blokhuis HJ (2004). Decision support system with semantic model to assess the risk of tail biting in pigs 1. Modelling. Appl Anim Behav Sci.

[bib1.bib15] Bracke MBM, Hulsegge B, Keeling L, Blokhuis H J (2004). Decision support system with semantic model to assess the risk of tail biting in pigs 2. Validation. Appl Anim Behav Sci.

[bib1.bib16] Bracke MBM, Zonderland JJ, Bleumer EJB (2007). Expert judgement on enrichment materials for pigs validates preliminary RICHPIG Model. Appl Anim Behav Sci.

[bib1.bib17] Bracke MBM, Edwards SA, Engel B, Buist WG, Algers B (2008). Expert opinion as “validation” of risk assessment applied to calf welfare. Acta Vet Scand.

[bib1.bib18] Bracke MBM, Edwards SA, Metz JHM, Noordhuizen JPTM, Algers B (2008). Synthesis of semantic modelling and risk analysis methodology applied to animal welfare. Animal.

[bib1.bib19] Bracke MBM, Koene P, Estevez I, Butterworth A, Jong I Cde (2019). Broiler welfare trade-off: A semi-quantitative welfare assessment for optimised welfare improvement based on an expert survey. PLoS One.

[bib1.bib20] Bracke MBM, Boumans IJMM, Nijland HJ, Bokkers EAM (2023). Review: Connecting circularity to animal welfare calls for a “novel” conceptual framework based on integrity. Animal.

[bib1.bib21] Chidgey KL (2023). Review: Space allowance for growing pigs: animal welfare, performance and on-farm practicality. Animal.

[bib1.bib22] Chowdhary KR, Chowdhary KR (2020). Fundamentals of Artificial Intelligence.

[bib1.bib23] Cooper R, Wemelsfelder F (2020). Qualitative behaviour assessment as an indicator of animal emotional welfare in farm assurance. Livestock.

[bib1.bib24] De Mol RM, Schouten W, Evers E, Drost H, Houwers H, Smits AC (2006). A computer model for welfare assessment of poultry production systems for laying hens. NJAS.

[bib1.bib25] EFSA (2007). Animal health and welfare aspects of different housing and husbandry systems for adult breeding boars, pregnant, farrowing sows and unweaned piglets – Scientific Opinion of the Panel on Animal Health and Welfare.

[bib1.bib26] EFSA (2010). Animal welfare risk assessment guidelines on housing and management (EFSA Housing Risk).

[bib1.bib27] EFSA (2012). Scientific Opinion on the welfare of cattle kept for beef production and the welfare in intensive calf farming systems.

[bib1.bib28] EFSA (2015). Scientific Opinion on welfare aspects of the use of perches for laying hens.

[bib1.bib29] EFSA (2023). Welfare of laying hens on farm.

[bib1.bib30] European Commission (2022). Study on animal welfare labelling: Final report.

[bib1.bib31] European Commission Eurobarometer shows how important Animal Welfare is for Europeans.

[bib1.bib32] Folkedal O, Pettersen JM, Bracke MBM, Stien LH, Nilsson J, Martins C, Breck O, Midtlyng PJ, Kristiansen T (2016). On-farm evaluation of the Salmon Welfare Index Model (SWIM 1.0): Theoretical and practical considerations. Anim Welfare.

[bib1.bib33] Heinonen M, Peltoniemi O, Valros A (2013). Impact of lameness and claw lesions in sows on welfare, health and production. Livest Sci.

[bib1.bib34] Helge Stien L, Noble C, Madaro A, Nilsson J, Alvestad R, Gismervik K, Harasimczuk E, Bleie H, Skaar Amthor K, Bracke MBM (2025). Reviewing the Severity of Handling-Induced Injuries in the LAKSVEL Protocol for Salmon Welfare. Rev Aquacult.

[bib1.bib35] Jones S, Neville V, Higgs L, Paul ES, Dayan P, Robinson ESJ, Mendl M (2018). Assessing animal affect: an automated and self-initiated judgement bias task based on natural investigative behaviour. Sci Rep.

[bib1.bib36] Kauselmann K, Krause ET, Glitz B, Gallmann E, Schrade H, Schrader L (2021). Effect of plant-based enrichment materials on exploration in rearing and fattening pigs (*Sus scrofa domesticus*). Appl Anim Behav Sci.

[bib1.bib37] Keeling LJ, Marier EA, Olmos Antillón G, Blokhuis HJ, Staaf Larsson B, Stuardo L (2022). A global study to identify a potential basis for policy options when integrating animal welfare into the UN Sustainable Development Goals. Front Anim Sci.

[bib1.bib38] Kirkden RD, Pajor EA (2006). Using preference, motivation and aversion tests to ask scientific questions about animals' feelings. Appl Anim Behav Sci.

[bib1.bib39] Neuhauser J, Hintze S, Rault JL, Smith S, Sirovnik J (2023). Training for a cognitive judgement bias task does not affect fear or telomere shortening in laying hens. Appl Anim Behav Sci.

[bib1.bib40] Pettersen JM, Bracke MB, Midtlyng PJ, Folkedal O, Stien LH, Steffenak H, Kristiansen TS (2014). Salmon welfare index model 2.0: an extended model for overall welfare assessment of caged Atlantic salmon, based on a review of selected welfare indicators and intended for fish health professionals. Rev Aquacult.

[bib1.bib41] Pinheiro Machado Filho LC, Teixeira DL, Weary DM, von Keyserlingk M, Hötzel MJ (2004). Designing better water troughs: dairy cows prefer and drink more from larger troughs. Appl Anim Behav Sci.

[bib1.bib42] Qin L, Chen Q, Feng X, Wu Y, Zhang Y, Li Y, Li M, Che W, Yu PS (2024). Large language models meet nlp: A survey [preprint]. arXiv.

[bib1.bib43] Rault JL, Bateson M, Boissy A, Forkman B, Grinde B, Gygax L, Harfeld JL, Hintze S, Keeling LJ, Kostal L, Lawrence AB, Mendl MT, Miele M, Newberry RC, Sandøe P, Špinka M, Taylor AH, Webb LE, Whalin L, Jensen MB (2025). A consensus on the definition of positive animal welfare. Biol Letters.

[bib1.bib44] Reimers N, Gurevych I (2019). Sentence-bert: Sentence embeddings using siamese bert-networks [preprint]. arXiv.

[bib1.bib45] Shimmura T, Bracke MBM, de Mol RM, Hirahara S, Uetake K, Tanaka T (2011). Overall welfare assessment of laying hens: comparing science-based, environment-based and animal-based assessments. Anim Sci J.

[bib1.bib46] Stien LH, Bracke MBM, Folkedal O, Nilsson J, Oppedal F, Torgersen T, Kittilsen S, Midtlyng PJ, Vindas MA, Øverli Ø, Kristiansen T S (2013). Salmon Welfare Index Model (SWIM 1.0): A semantic model for overall welfare assessment of caged Atlantic salmon: review of the selected welfare indicators and model presentation. Rev Aquacult.

[bib1.bib47] Tschirren L, Bachmann D, Güler AC, Blaser O, Rhyner N, Seitz A, Zbinden E, Wahli T, Segner H, Refardt D (2021). MyFishCheck: A Model to Assess Fish Welfare in Aquaculture. Animals.

[bib1.bib48] Ursinus WW, Schepers F, de Mol RM, Bracke MBM, Metz J, Groot Koerkamp P (2009). COWEL: a decision support system to assess welfare of husbandry systems for dairy cattle. Anim Welfare.

[bib1.bib49] Verdon M (2022). A review of factors affecting the welfare of dairy calves in pasture-based production systems. Anim Prod Sci.

[bib1.bib50] Vonholdt-Wenker ML, Benthin J, Kauselmann K, Bracke MBM, Krause ET (2026). Semantic modelling of animal welfare explained – Part 2: The basis of welfare weighting and usage of scientific information. Arch Anim Breed.

[bib1.bib51] Zhang C, Da Silva Torres R, Gansel L, Bracke M (2026). Automated extraction of scientific statements for integrated assessment of animal welfare using Language models. Smart Agric Technol.

