# Supplementary material for: Semantic modelling of animal welfare explained – Part 1: Calculating overall welfare scores for husbandry systems using the ANyWEL model framework
Source: Arch Anim Breed. 2026 Jul 16;69(3):363–82. doi: 10.5194/aab-69-363-2026 (PMC13480338; doi:10.5194/aab-69-363-2026)
Supplement: Supplementary file 1 [file aab-69-363-2026-supplement.zip › aab-69-363-2026-supplement-title-page.pdf]

Supplement of Arch. Anim. Breed., 69, 363–382, 2026
https://doi.org/10.5194/aab-69-363-2026-supplement
© Author(s) 2026. CC BY 4.0 License.

Open Access
Archives Animal Breeding

Supplement of

Semantic modelling of animal welfare explained – Part 1: Calculating overall welfare scores for husbandry systems using the ANyWEL model framework

Janine Benthin et al.

Correspondence to: Janine Benthin (janine.benthin@fli.de)

- aab-69-363-2026-supplement-title-page.pdf
- Benthin et al Supplementary Material S1_R2.xlsx
- Benthin et al Supplementary Material S2_R2.xlsx

The copyright of individual parts of the supplement might differ from the article licence.
